# Supplementary material for: Dynalign II: common secondary structure prediction for RNA homologs with domain insertions
Source: Nucleic Acids Res. 2014 Nov 21;42(22):13939–48. doi: 10.1093/nar/gku1172 (PMC4267632; doi:10.1093/nar/gku1172)
Supplement: SUPPLEMENTARY DATA [file supp_gku1172_nar-02021-z-2014-File012.zip › manual/Text/bipartition.html]

RNAstructure Command Line Help: bipartition


|  |  |  |
| --- | --- | --- |
|  | RNAstructure Command Line Help  bipartition and bipartition-smp | - Contents - Index |
| bipartition is used to predict the partition function for structures containing two strands. Note that the structures do not include those with intramolecular pairs. Note that output is written to a partition function save file where the sequences are concatenated, with an intermolecular linker between them ("III"). Note also that the partition function save file remembers the nucleic acid type it was built with (RNA or DNA), so any further calculations with that file will assume they are done with that same nucleic acid type. bipartition-smp is a parallel processing version for use on multi-core computers, built using OpenMP. USAGE: bipartition <seq file 1> <seq file 2> <pfs file> [options] OR: bipartition-smp <seq file 1> <seq file 2> <pfs file> [options] Required parameters: <seq file 1> \| The name of a sequence file containing input data for the first sequence. Note that lowercase nucleotides are forced single-stranded in structure prediction.; <seq file 2> \| The name of a sequence file containing input data for the second sequence. Note that lowercase nucleotides are forced single-stranded in structure prediction.; <pfs file> \| The name of a binary partition function save file to which output will be written. Options which don't require added values: -d, -D, --DNA \| Specify that the sequence is DNA, and DNA parameters are to be used. Note that the backbone type must be the same for both sequences. Default is to use RNA parameters.; -h, -H, --help \| Display the usage details message. Options which require added values: -t, -T, --temperature \| Specify the temperature at which calculation takes place in Kelvin. Default is 310.15 K, which is 37 degrees C. Notes for smp: bipartition-smp, by default, will use all available compute cores for processing. The number of cores used can be controlled by setting the OMP\_NUM\_THREADS environment variable. References: 1. Reuter, J.S. and Mathews, D.H. "RNAstructure: software for RNA secondary structure prediction and analysis." *BMC Bioinformatics*, 11:129. (2010). 2. Lu, Z.J. and Mathews, D.H. "Efficient siRNA Selection Using Hybridization Thermodynamics." *Nucleic Acids Res.*, 36:640-647. (2007). | | |
| Visit The Mathews Lab RNAstructure Page for updates and latest information. | | |
